# Somatic Symptoms in the German General Population from 1975 to 2013

**DOI:** 10.1038/s41598-020-58602-6

**Published:** 2020-01-31

**Authors:** Manfred E. Beutel, Eva M. Klein, Michaela Henning, Antonia M. Werner, Juliane Burghardt, Ana Nanette Tibubos, Gabriele Schmutzer, Elmar Brähler

**Affiliations:** 1 Department of Psychosomatic Medicine and Psychotherapy, University Medical Center Mainz, Johannes Gutenberg University Mainz, Mainz, Germany; 2 Department of Psychosomatics and Psychotherapy, University Hospital Cologne, University of Cologne, Cologne, Germany; 3 Department of Medical Psychology and Medical Sociology, University Medical Center Leipzig, University of Leipzig, Leipzig, Germany

**Keywords:** Epidemiology, Risk factors, Signs and symptoms

## Abstract

The study determines how burden and patterns of somatic symptom reporting developed over almost four decades in the general German population. Additionally, we studied how socio-demographic factors affected the degree of somatic symptoms. Population-based samples representative for West Germany between 18 and 60 years of age were analyzed comparing three cross-sectional samples of 1975 (*N* = 1601), 1994 (*N* = 1416), and 2013 (*N* = 1290) by conducting a three-way analysis of variance (sex, age, survey). The prevalence rates for somatic symptoms in men and women were lower in the more recent surveys; this affected women most strongly. Exhaustion and musculoskeletal complaints remained leading symptoms (affecting 25%, resp. 11% of the men and 30%, resp. 19% of the women). There was a slight increase in women’s prevalence of exhaustion from 1994 (15%) to 2013 (19%). As determined by stepwise multiple regression, somatic symptoms were consistently associated with female sex and higher age. In the 2013 survey, education became an additional negative predictor of somatic symptom load, while the impact of age and sex on somatic symptoms reporting decreased. Somatic symptoms remain a major burden in the general population. Findings are interpreted with regard to improved living and health care conditions, different cohort experiences, and more public health information.

## Introduction

A high burden of somatic symptoms has been associated with considerable suffering, health care utilization and costs. Widespread somatic symptoms not only indicate somatic illnesses, but also common mental disorders such as depression^1–5^. Somatic symptom burden has been associated with female sex^6,7^, higher age^8,9^, lower education^8,10^ and socio-economic status^7^, and disruption of relationships by separation, divorce or widowhood^10^. Shaped by culturally bound factors such as illness models, somatic awareness, and interoceptive accuracy, somatic symptom patterns and burdens differed between cultural and ethnic groups^11^.

Changes of psychiatric classification^12,13^ and social context may alter somatic symptom reporting^14^ along with media coverage of health risks and iatrogenic factors^15^. As studies have mostly been cross-sectional and assessment methods have changed^16^, little is known about trends of somatic symptoms in the general population over time^10^. While numerous international studies and the growing awareness of mental health tend to create the impression that we are facing a surge of mental disorders, evidence is contradictory. According to the systematic review by Wittchen *et al*. (2011), an overall incline of mental disorders in Europe was due to inclusion of additional diagnoses. Somatoform disorders remained constant (6.3% in 2005 and 4.9% in 2011)^17^.

Based on a unique data set from representative surveys in West Germany, we analyzed changes in major somatic complaints in three cross-sectional samples with participants aged between 18 and 60 years (birth cohorts from 1914 to 1995) gathered in the years 1975, 1994 and 2013. Data cover a period of substantial political (e.g. German reunification in 1990), demographic (e.g. aging population, declining gaps in women’s education) and public health (e.g. obesity epidemic, expanding mental health care) changes. While drawn independently, each sample was recruited by using comparable procedures and was evaluated by the same criteria.

We inquired, how somatic symptom patterns and burdens of women and men shifted over time, and across the age range from 18 to 60 years. The Giessen Subjective Complaints List (GBB-8^3^) assessed four of the most frequently measured dimensions of somatic complaints with two different symptoms: cardiovascular (palpitation and dizziness), gastrointestinal (abdominal feeling of fullness/pressure and stomachache), musculoskeletal (back/sacroiliac pain and neck/shoulder pain), and exhaustion symptoms (tiredness and exhaustibility). We looked for symptom reporting for each symptom separately and the total score. Above that, based on a predefined cut-off score (total score > 12), we determined high somatic symptom burden in women and men over time. Analyzing each survey sample separately, we included the demographic variables sex, age, and education to predict symptom burden.

## Results

### Study participants

As Table 1 shows, comparable numbers of participants were recruited in all three samples, with a slight preponderance of women. Corresponding to general trends in the German population, the age distribution gradually shifted toward older participants. In 1975, 72% reported being married and living together. The proportion living with a partner further declined from 64% in 1994 to 53% in 2013 (χ²(2) = 33.41, *p* < 0.001). There was a strong increase in education; overall A-level (the German *Abitur*) more than doubled (χ²(2) = 89.23, *p* < 0.001), with women increasingly catching up with men. Also, unemployment increased from 1.7% to 7.3% (χ²(2) = 54.15, *p* < 0.001).

**Table 1:** Demographic Data of the Samples.

	1975 *N* = 1601	1994 *N* = 1416	2013 *N* = 1290
No. (%) of women	862 (53.8)	807 (56.9)	322 (53.3)
No. (%) of age range 18–30 years	469 (29.3)	410 (29.0)	260 (25.0)
No. (%) of age range 31–40 years	466 (29.1)	392 (27.7)	361 (20.2)
No. (%) of age range 41–50 years	333 (20.7)	288 (20.3)	347 (28.0)
No. (%) of age range 51–60 years	333 (20.7)	326 (23.0)	322 (26.9)
No. (%) of partnership Total	1145 (72.2)^a^	908 (64.1)^b^	686 (53.2)^b^
No. (%) of partnership in women	622 (72.8)^a^	534 (66.2)^b^	366 (53.3)^b^
No. (%) of partnership in men	523 (72.0)^a^	374 (61.4)^b^	320 (53.1)^b^
No. (%) of education A-level total	162 (10.2)	257 (18.2)	298 (23.1)
No. (%) of education A-level in women	52 (6.1)	113 (14.0)	149 (21.7)
No. (%) of education A-level in men	110 (15.0)	144 (23.7)	149 (24.8)
No. (%) of unemployed total	27 (1.7)	65 (4.6)	94 (7.3)
No. (%) of unemployed women	13 (1.5)	34 (4.2)	43 (6.3)
No. (%) of unemployed men	14 (1.9)	31 (5.1)	51 (8.5)

*Note*. ^a^Married, living together; ^b^Living with a partner.

### Somatic complaints over time

Table 2 shows the percentages of men and women suffering from each of the eight somatic symptoms at least to a moderate degree.

**Table 2:** Men and women burdened by symptoms over time.

Symptom	Year	Total Sample	Men	Women
		%^*a*^ [95% CI]	%^*a*^ [95% CI]	% ^*a*^ [95% CI]
back/sacroiliac pain	1975	39.7 [39.67–39.73]	32.1 [33.04–32.16]	46.2 [46.14–46.26]
	1994	35.8 [35.77–35.83]	36.0 [35.93–36.07]	35.7 [35.64–35.76]
	2013	27.5 [27.47–27.53]	24.5 [24.44–24.56]	30.4 [30.34–30.46]
tiredness	1975	37.3 [37.27–37.33]	27.6 [27.55–27.65]	45.6 [45.54–45.66]
	1994	20.1 [20.08–20.12]	17.6 [17.55–17.65]	21.9 [21.86–21.94]
	2013	21.9 [21.87–21.93]	16.1 [16.06–16.14]	26.6 [26.54–26.66]
neck/shoulder pain	1975	26.0 [25.98–26.02]	18.5 [18.46–18.54]	32.6 [32.55–32.65]
	1994	31.7 [31.67–31.73]	28.4 [28.33–28.47]	34.2 [34.15–34.25]
	2013	21.9[21.87–21.93]	16.7 [16.65–16.75]	26.2 [26.14–26.26]
palpitation	1975	25.5 [25.48–25.52]	18.4 [18.36–18.44]	31.7 [31.65–31.75]
	1994	10.4 [10.39–10.41]	8.2 [8.18–8.22]	12.0 [11.97–12.03]
	2013	7.1 [7.09–7.11]	6.1 [6.08–6.12]	7.9 [7.88–7.92]
dizziness	1975	20.4 [20.38–20.42]	13.1 [13.07–13.13]	26.7 [26.66–26.74]
	1994	10.5 [10.49–10.51]	6.7 [6.68–6.72]	13.3 [13.27–13.33]
	2013	5.7 [5.69–5.71]	3.7 [3.69–3.71]	7.4 [7.38–7.42]
exhaustibility	1975	19.5 [19.48–19.52]	12.7 [12.67–12.73]	25.4 [25.36–25.44]
	1994	13.5 [13.48–13.52]	11.0 [10.97–11.03]	15.4 [15.37–15.43]
	2013	15.3 [15.28–15.32]	10.9 [10.87–10.93]	19.1 [19.06–19.14]
abdominal feeling of fullness/pressure	1975	21.0 [20.98–21.02]	19.3 [19.26–19.34]	22.4 [22.36–22.44]
	1994	15.1 [15.08–15.12]	13.5 [13.46–13.54]	16.4 [16.37–16.43]
	2013	9.6 [9.59–9.61]	8.0 [7.98–8.02]	11.1 [11.07–11.13]
stomachache	1975	14.4 [14.38–14.42]	13.7 [13.67–13.73]	15.0 [14.97–15.03]
	1994	11.8 [11.79–11.81]	11.2 [11.17–11.23]	12.3 [12.27–12.33]
	2013	9.2 [9.19–9.21]	6.81 [6.79–6.83]	11.2 [11.17–11.23]

*Note*. ^a^Moderately, strongly, very strongly; CI = 95% confidence intervals.

In 1975, 1994, and 2013 the three most frequent complaints were back/sacroiliac pain, neck/shoulder pain, and tiredness. While back pain and tiredness affected almost half of the women (46.2%, 45.6% respectively) and almost one third (32.1%, 27.6%, respectively) of the men in 1975, the percentage of men and women reporting moderate to very strong complaints decreased in the later surveys. Furthermore, in 1975, neck/shoulder pain affected almost one in three women, and over 20% suffered from palpitations (31.7%), dizziness (26.7%), exhaustibility (25.4%) and abdominal pressure (22.4%); stomachache was lowest at 15%. Almost 20% of the men reported abdominal pressure, neck pain, and palpitations; stomachache, dizziness, and exhaustibility were lower (12.7 to 13.7%). All these somatic symptoms were less troubling in the following survey samples, but to different degrees: Compared to 1975, in 2013 palpitations and dizziness were lower by two thirds and abdominal discomfort by more than half alongside with stomachache in men. Back pain was the most frequent symptom in 2013, reported by 24.5% of the men and 30.4% of women, followed by neck pain (16.7% vs. 26.2%), tiredness (16.1% vs. 26.7%) and exhaustion (10.9% vs. 19.1%).

**Figure 1:**
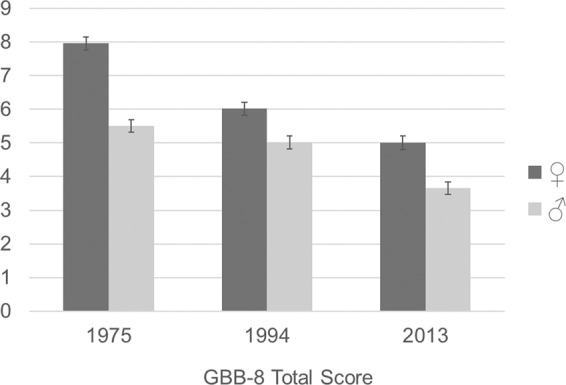
Total somatic symptoms of women and men over time. *Note*. Gießen Subjective Complaint List-8 (GBB-8). Overall means and standard errors are presented for men and women separately.

Figure 1 shows the total symptom burden (range 0 to 32) as overall mean scores of men and women. Men reported an overall symptom severity of 5.5 (*SD* = 5.1) in 1975, 5.0 (*SD* = 4.8) in 1994, and 3.7 (*SD* = 4.5) in 2013. Women indicated as mean overall symptom severity 8.0 (*SD* = 5.8) in 1975, 6.0 (*SD* = 5.4) in 1994, and 5.0 (*SD* = 5.3) in 2013. The three-way Analysis of Variance (ANOVA) with the factors sex, age group and year of survey confirmed higher overall scores of women compared to men (*p* < 0.001; medium effect size; η^2^ = 0.02) and small age effects with higher scores of older versus younger age groups (*p* < 0.01; η^2^ = 0.05). Symptoms were lower at subsequent surveys (*p* < 0.01; η^2^ = 0.04). However, there was only one very small significant interaction of sex and survey indicating that the mean differences were slightly bigger in women than in men (*p* < 0.01; η^2^ = 0.003).

Lower symptom load is also illustrated in Supplementary Table 1 by the lower proportions of high scorers (GBB-8 > 12): In women, the proportion was initially 21% in 1975 dropping to 13% in 1994 and finally to 11% in 2013. Men started out with a lower proportion of high scorers in 1975 at 10% which was only slightly lower in 1994 (9%) and considerably lower at 6% in 2013.

**Figure 2:**
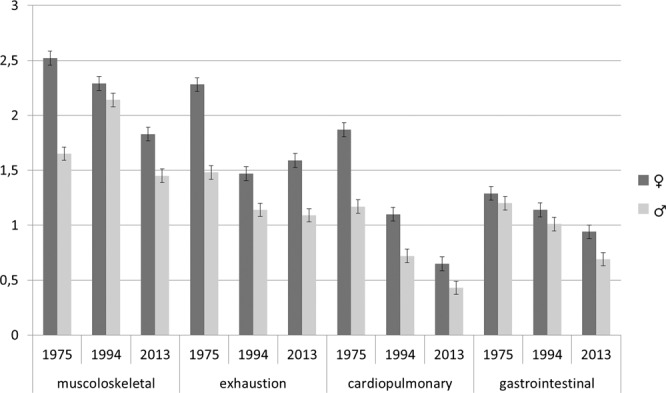
Dimensions of somatic symptoms over time according to the GBB-8: Women and men. *Note*: Means and standard errors are presented; GBB-8 = Gießen Subjective Complaint List 8; range: 0–4.

Figure 2 shows the mean scores and standard errors of the four dimensions of somatic complaints, separately for men and women over time in declining order across the three surveys. Except for an increase of musculoskeletal complaints in men in 1994 and exhaustion in women in 2013, somatic symptoms declined across all four domains in men and in women.

Further ANOVAs revealed main effects for sex, age group, and survey year in all four domains with women and older participants reporting higher scores. Also, scores in the 1975 survey were higher than in the 1994 and 2013 surveys (scores of 1975 > 1994 > 2013). Significant, yet very small interactional effects indicated that declines of symptom reporting were stronger for women than men regarding musculoskeletal (*p* < 0.01; η^2^ = 0.004) and cardiopulmonary problems (*p* = 0.001; η^2^ = 0.003), and exhaustion (*p* = 0.004; η^2^ = 0.003), but not for gastrointestinal complaints. An interaction between age group and cohort was found for cardiopulmonary complaints (*p* = 0.002; η^2^ = 0.01) indicating less symptom reporting for all age groups in the 2013 cohort. Furthermore, a three-way interaction of sex by age group by survey was significant indicating an increase of exhaustion complaints comparing the 1994 and 2013 cohort in women aged 41 to 50 and 51 to 60 years (*p* = 0.031; η^2^ = 0.003).

### Predictive Factors for Total Somatic Symptom Load

Table 3 shows predictors of total symptom load for the three surveys.

**Table 3:** Results of Stepwise Multivariate Regression predicting Somatic Symptom Load (Total Score).

	1975	1994	2013
Step 1			
Age, coefficient (95% CI)	0.11*** (0.09; 0.13)	0.09*** (0.07; 0.11)	0.09*** (0.06; 0.11)
R^2^	0.058***	0.042***	0.042***
Adjusted *R*^2^	0.057***	0.042***	0.041***
Step 2			
Sex, coefficient (95% CI)	2.19*** (1.66; 2.72)	0.94*** (0.41; 1.47)	1.42*** (0.89; 1.95)
Age, coefficient (95% CI)	0.10*** (0.08; 0.12)	0.09*** (0.07; 0.11)	0.09*** (0.07; 0.11)
R^2^	0.095***	0.051***	0.062***
Adjusted *R*^2^	0.094***	0.049***	0.060***
Step 3			
Sex, coefficient (95% CI)	NA	NA	1.40*** (0.86; 1.95)
Age, coefficient (95% CI)	NA	NA	0.86*** (0.83; 0.88)
Education, coefficient (95% CI)	NA	NA	−0.76* (−1.39; −0.13)
R^2^	NA	NA	0.066***
Adjusted *R*^2^	NA	NA	0.064***

*Note*. NA = Step 3 was not applicable as education was no significant predictor in stepwise regression. ***p < 0.001; *p < 0.05.

As Table 3 shows, we found similar patterns of predictors: Age was a factor in all three surveys as well as female sex which both were associated with higher symptom load in 1975, 1994 and 2013. On a descriptive level, the explained variance of somatic symptom load through age became smaller over the three surveys, while sex had a stronger impact in 1975 vs. 1994 and 2013. Education was only a significant negative predictor in 2013. Overall, the proportions of explained variance in somatic symptom load by sex and age were higher in 1975 (9.4%) than in 1994 (4.9%) and 2013 (6.0%). When including education as a predictor, explained variance for somatic symptom load increased slightly to 6.4% in 2013.

## Discussion

Somatic symptom load has declined considerably in women and men living in the western states of Germany (former Federal Republic of Germany) over almost four decades, from 1975 to 2013. Interestingly, the decline was stronger in women than in men, except for gastrointestinal complaints. Women had reported considerably more somatic symptoms in 1975, exceeding men’s ratings by 50% and more. In 2013, the degree of somatic symptoms reported by women still exceeded men’s ratings, but women’s intensity and pattern of symptom reporting more closely resembled men’s reporting. Musculoskeletal and exhaustion complaints remained the leading symptoms, burdening one in four (back pain), respectively one in six men (tiredness, neck pain). Back pain and tiredness, respectively, bothered almost one in three women and one in four women was affected by neck pain. As opposed to the trend, there was an increase of exhaustion symptoms in women in the age groups of 41 to 50 and 51 to 60 years from 1994 to 2013.

In multivariate regression analyses, predictors of somatic symptom burden were consistent with previous findings^10^: Female sex and age were consistent predictors in 1975, 1994, and 2013. However, consistent with the descriptive findings, the contribution of sex and age declined across surveys. Education was a negative predictor of symptom load only in 2013. Observed changes raise issues about living conditions and public health changes related to cohort and gender.

While there was considerable overlap, each survey was composed of different birth cohorts with different patterns of socialization. Notably, in 1975 the majority of participants, born between 1915 and 1944, had experienced the second (some even the first) world war in their childhood, adolescence or young adulthood. This applied to none of the 2013 participants (with the 1994 participants in between). As Supplementary Table 1 illustrates, age effects levelled out in women and in men across surveys. The war generations had by far the greatest proportions of high scorers in men and women. Thus, stronger age effects in the earlier surveys may also have reflected long-term sequelae of war experience with associated threats, losses and periods of starvation and malnutrition^18,19^. Interestingly, overall declines of symptom load took place despite aging in the population with an increasing proportion of the age group from 51 to 60 years. As younger post-war generations were raised in prosperity, improvements of subjective health and disability-free life expectancy may have decreased symptom reporting in the age range studied.

The proportion of A-level education more than tripled among women over time, so that they reached a level comparable to men. There was a strong decline of men and women living in a partnership, which had often followed the traditional gender roles of “housewife” vs. “breadwinner” in the 1970s. Over the past decades, women have occupied more full-time and part-time jobs. For instance, the relation between women’s to men’s wages (i.e., unadjusted wage gap) increased in Germany from 1950 to 2004 from 55% to 71%^20^. As women gained financial and economic independence, increasing similarity of women’s and men’s living and working conditions may have diluted gender role expectations proscribing higher willingness to concede symptoms on behalf of men vs. women and contributed to greater similarities of somatic symptom reporting^10,21^.

With the rapid development of the internet, information and communication technologies accelerated social change radically, altering work conditions and careers, education, mobility, and social relationships^22^. The recent increase of exhaustion in women concurs with increased reports of insomnia in the general population^23,24^. Increasing fatigue in women may be related to strains of combining work and motherhood and the increase of single parenting, both less prominent in the 1970s^25^. For men and women, fatigue has been related to an increasing communication load, the need to be always online or the fear of missing out text messages, social networks^26^, and to bed time use of electronic media and mobile phone e.g.,^27^.

Declines in cardiovascular and gastrointestinal complaints may have been promoted by medical developments, especially improved treatment options for cardiovascular diseases and gastric ulcers. A weak heart, low blood pressure or poor circulation have been described as culture-specific somatic symptoms in Germany, and illness models have been affected by social feedback models signaling the seriousness of a condition^11^. Since the mid-1970s, psychotherapy has been increasingly reimbursed by the public health insurance in Germany, and new psychosomatic hospitals and rehabilitation clinics have been established in order to provide health care for mental, including somatization disorders^28^. Increased acceptance of psychotherapeutic help-seeking and referral^29^, and improved mental health knowledge and care may have also decreased reporting of distress by somatic symptoms. As indicated by the 2013-survey, higher education has contributed to the reduction of somatic symptom reporting^30^.

The strong decline of high scorers by 50% in women and one third in men contradicts popular notions of increasing psychosomatic symptoms. Given demographic aging with more overweight and obesity in the population^31^ and the higher proportion of individuals living alone, we could have expected an increase of somatic symptom reporting instead of a decrease. The only observed relevant increase of symptoms emerged for women between 40 and 60 years who reported more exhaustion in 2013 than 1994.

Repeated cross-sectional assessments with independent samples from the same population have been strongly advocated to detect variations of cultural conceptions of illness over time periods, which could not be covered longitudinally due to shifts of sample composition and attrition^32^. Major strengths of our study are repeated assessments by comparable sampling procedures and an identical standardized questionnaire over the long timespan enabling us to identify the time trends of symptom reporting in non-medical populations. While we sampled the same German states, we are aware that we could not assess the impact of migration from other countries and from the Eastern states of Germany following reunification. Based on our measure we cannot differentiate between acute and chronic somatic symptom burden. The cut-off score used requires further validation.

In summary, results demonstrate the malleability of patterns of symptom reporting in the general population and its associations with important factors such as sex, age, cohort, the context of the public health system, and education. These factors should inform medical care. For example, somatic symptoms should not be inquired in isolation, but tend to cluster (e.g. fatigue, musculoskeletal, gastrointestinal, cardiovascular symptoms). Even if relationships may vary (e.g. decline of sex effects, higher relevance of aging in women), female sex, higher age and educational level/knowledge about health issues need to be taken into account in evaluating somatic symptom reporting.

## Methods

### Study design

Infratest conducted the survey in 1975, with 1,601 participants. In 1994 and 2013, USUMA surveyed participants, with 1,416 included cases in 1994 and 1,290 in 2013. Households were selected by the random-route-procedure; the target person in each household was also selected randomly. The representativeness of the study was confirmed by samples of the recognized German market research institutes association *Arbeitskreis Deutsche Marktforschungsinstitute* (ADM). All surveys were conducted in accordance with the Declaration of Helsinki, and they fulfilled the ethical guidelines of the International Code of Marketing and Social Research Practice of the International Chamber of Commerce and of the European Society of Opinion and Marketing Research. The Ethics Committee of the Medical Department of the University of Leipzig approved the studies of 1994 and 2013. The response rates amounted to 75% in the sample of 1975, 65% in 1994, and 57.5% in 2013. A decline in response rates over the last decades has been observed in other survey studies as well^33,34^. In order to ensure comparability with the previous one, the two latter samples after the German reunification included only participants living in the Western states of Germany and within the age range of 18 to 60 years. Data-collection took place in the participants’ homes, after informed consent has been given. First, socio-demographic data was obtained by face-to-face interviews. Second, participants completed a questionnaire, containing self-report measures for many different health-related variables, including the Giessen Subjective Complaints List (GBB-8^3^).

### Measures

#### Somatic symptoms

The Giessen Subjective Complaints List with eight items (GBB-8^3^) is a popular and well-established self-report questionnaire for the assessment of subjective health complaints in German speaking countries and it was used in all of the three surveys. It derived from the highly reliable GBB-24^35^ with originally 24 health complaints. The brief version with eight items covers back/sacroiliac pain, neck/shoulder pain, palpitation, dizziness, tiredness, exhaustibility, abdominal feeling of fullness/pressure and stomach ache which are rated on a Likert scale ranging from 0 = *not at all*, 1 = *hardly*, 2 = *moderately*, 3 = *considerably* to 4 = *strongly*, indicating how troubling each complaint is perceived. The eight items constitute four scales, each consisting of two items and good internal consistency (Cronbach’s Alpha): exhaustion (α = 0.83), gastrointestinal complaints (α = 0.68), musculoskeletal (α = 0.85), and cardiovascular complaints (α = 0.68). Confirmatory factor analysis supported the four-factorial model^3^. Correlation coefficients of the brief and the long form were between *r* = 0.86 and *r* = 0.95 (sum score)^3^. We built scores for each scale and a total score of the GBB-8. For the total score we used a cut-off score greater than 12 in order to define a high somatic symptom burden corresponding to the cut-off score of the Somatic Symptom Scale (SSS-8^2^) which has the same response format as the GBB-8.

#### Demographic data

Sex, education, and unemployment were assessed with identical items in all cohorts. For partnership, we ascertained “married, living together” in 1975, from 1994 onward instead it was asked: “Do you live together with your partner?”.

### Statistical analysis

We calculated means and standard deviations, respectively standard errors for total scores and the four domains of somatic symptoms. Somatic symptoms are compared across surveys by means via Analysis of Variance (ANOVA) with the three factors sex (women, men), age group (in years: 18–30, 31–40, 41–50, 51–60) and survey year (1975, 1994, 2013). As effects sizes, we reported partial Eta-squared with η² = 0.01 indicating a small, and η² = 0.06 indicating a medium effect^36^. Additionally, we present the burden of individual symptoms of at least moderate degree and the proportions of participants with a high symptom load over time and separately for men and for women, including 95% confidence intervals. Predictors of somatic symptom load (total symptoms) were separately computed via multiple stepwise regression analyses for each survey with sex, age, and education as predictors. Statistics were computed by SPSS for Windows release 6.1 or SPSS 24.

## Supplementary information

Supplementary information

## Data Availability

The datasets generated during and/or analyzed during the current study are available from the corresponding author on reasonable request.
