# Supplementary material for: Somatic Symptoms in the German General Population from 1975 to 2013
Source: Sci Rep. 2020 Jan 31;10:1595. doi: 10.1038/s41598-020-58602-6 (PMC6994459; doi:10.1038/s41598-020-58602-6)
Supplement: Supplementary file 1 — Supplementary information [file 41598_2020_58602_MOESM1_ESM.pdf]

Somatic Symptoms in the German General Population from 1975 to 2013

Manfred E. Beutel, MD[1]; Eva M. Klein, Dipl.-Psych.[1]; Michaela Henning, MD[1,2]; *Antonia M. Werner, Dipl.-Psych.[1]; Juliane Burghardt, PhD[1]; Ana Nanette Tibubos, PhD[1]; Gabriele Schmutzer, Dipl.-Math.[3]; Elmar Brähler, PhD [1,3]

[1] Department of Psychosomatic Medicine and Psychotherapy, University Medical Center Mainz, Johannes Gutenberg University Mainz, Germany

[2] Department of Psychosomatics and Psychotherapy, University Hospital Cologne, University of Cologne, Germany

[3] Department of Medical Psychology and Medical Sociology, University Medical Center Leipzig, University of Leipzig, Germany

***Corresponding Author:** Antonia M. Werner, Dipl.-Psych., Dept. of Psychosomatic Medicine, University Medical Center Mainz, Untere Zahlbacher Str. 8, 55131 Mainz, Germany (Antonia.Werner@unimedizin-mainz.de).

Supplementary Information

Supplementary Table 1. Proportions of women and men with a high symptom load over age and time.

	Women			Men		
Survey year	1975	1994	2013	1975	1994	2013
	GBB-8 > 12 (%)			GBB-8 > 12 (%)		
18-30 yrs.	14.29	10.92	10.29	6.75	6.98	4.76
31-40 yrs.	19.65	8.92	5.04	5.17	8.38	5.79
41-50 yrs.	24.72	12.50	9.85	12.34	7.03	7.59
51-60 yrs.	25.93	19.39	17.65	21.74	16.05	5.08
Overall	20.84	12.76	10.92	9.76	9.36	5.80

Note: GBB-8 = Gießen Subjective Complaint List-8.
